# Neuropsychological deficits in patients with persistent COVID-19 symptoms: a systematic review and meta-analysis

**DOI:** 10.1038/s41598-023-37420-6

**Published:** 2023-06-26

**Authors:** Saioa Sobrino-Relaño, Yolanda Balboa-Bandeira, Javier Peña, Naroa Ibarretxe-Bilbao, Leire Zubiaurre-Elorza, Natalia Ojeda

**Affiliations:** Department of Psychology, Faculty of Health Sciences, University of Deusto, Av. de Las Universidades, 24, 48007 Bilbao, Spain

**Keywords:** Signs and symptoms, Diseases

## Abstract

Long-term persistent symptoms of COVID-19 affect 30–80% of patients who have recovered from the disease and may continue for a long time after the disease has been overcome. The duration of these symptoms over time might have consequences that affect different aspects of health, such as cognitive abilities. The main objective of this systematic review and meta-analysis was to objectify the persistent COVID-19 cognitive deficits after acute phase of infection and to summarize the existing evidence. Additionally, we aimed to provide a comprehensive overview to further understand and address the consequences of this disease. Our protocol was registered in PROSPERO (CRD42021260286). Systematic research was conducted in the Web of Science, MEDLINE, PubMed, PsycINFO, Scopus, and Google Scholar databases from January 2020 to September 2021. Twenty-five studies were included, six of which were analyzed for the meta-analysis, and consisted of 175 patients who had recovered from COVID-19 and 275 healthy individuals. Analyses of cognitive performance of post-COVID-19 patients and healthy volunteers were compared using a random-effects model. The results showed an overall medium–high effect size (*g* = −.68,* p* = .02) with a 95% CI (−1.05 to −.31), with a significantly moderate level of heterogeneity among studies (*Z* = 3.58, *p* < .001; *I*^2^ = 63%). The results showed that individuals who had recovered from COVID-19 showed significant cognitive deficits compared to controls. Future studies should carefully assess the long-term progression of cognitive impairments in patients with persistent COVID-19 symptoms, as well as the effectiveness of rehabilitation interventions. Nevertheless, there is an urgent need to know the profile to speed up development of prevention plans as well as specific interventions. Since more information is being obtained and more studies are being conducted on the subject, the need to examine this symptomatology multidisciplinary to achieve greater scientific evidence of its incidence and prevalence has become increasingly clear.

## Introduction

At the end of December 2019, coronavirus disease 2019 (COVID-19) caused by the Severe Acute Respiratory Syndrome-CoV-2 (SARS-CoV-2) violently shook the health care system worldwide, affecting the entire population. COVID-19 has been defined by the World Health Organization (WHO) as an "infectious disease" characterized by symptoms such as fever, dry cough and fatigue, with also a high level of spread both by airborne droplets (respiratory secretions) and indirect contact with infected secretions^1^. In the severe acute phase of the disease, the most common symptoms include respiratory distress, cerebrovascular disorders and neurological symptoms (e.g. headache, dizziness, test and smell dysfunctions, etc.), among others^2–7^. Furthermore, the virus can cause both pulmonary and systemic inflammation, affecting multiple organs^2^. Indeed, COVID-19 is recognized as a multi-organ disease with a wide spectrum of manifestations^8–11^. Coherently with the notion that the central nervous system (CNS) could be both directly and indirectly involved in the acute phase of the infection, evidence has been reported about neurological manifestations, such as dizziness/vertigo, headache, hypogeusia, hyposmia, muscular, ischemic stroke, intracerebral hemorrhage, encephalopathy, and others^12–16^. Several studies have also identified an increasing number of different neuropsychiatric and neuropsychological alterations^17–20^, such as anxiety, mood alterations, agitation, confusion and delirium. It is also important to mention that these symptoms, with neuromuscular and cerebrovascular alterations, are much more frequent in older patients with severe infection or in the presence of multiple comorbidities^6,8–11,21^. Additionally, several studies have shown evidence of cognitive deficits in patients with COVID-19, both in those with mild symptoms and in those suffering from severe forms of disease and requiring intensive care unit (ICU) admission^18^. The effects of SARS-CoV-2 infection on cognition may be related to the indirect CNS involvement due to the virus infection, as well as to the infection damage on multiple system, to systemic inflammation, immune system dysregulation or hypoxia^22,23^. Alternatively, evidence about a direct infection of CNS had been also provided^5,6,8,16,21^.

In some studies conducted so far, 80% of patients presented neuropsychological deficits; the most common being: visuospatial and executive functions^17,18^, working memory^17,19^, abstraction ability^17,19^ and orientation^17^ according to the Montreal Cognitive Assessment (MoCA) test, among others.

These symptoms can extend beyond the acute stage of COVID-19 infection^24–29^. The WHO has referred to this phenomenon as post-COVID-19 and has defined it as "*a condition that occurs in individuals with a probable or confirmed history of SARS-CoV-2 infection, usually 3 months from the onset of COVID-19 with symptoms that last for at least 2 months and cannot be explained by an alternative diagnosis*"^30^. Additionally, this definition includes common symptoms that impact daily life, such as fatigue, shortness of breath, and cognitive dysfunction^19,24,30–32^. In this review, we will use the terms “post-acute COVID-19 syndrome” and “persistent COVID symptoms.”

Due to the relatively short time since the outbreak of the pandemic, more data is needed on the actual consequences of the disease on the cognitive functions of patients with persistent COVID symptoms (especially in patients who did not require hospitalization), as well as on the uncertain impact of post-acute COVID on respiratory function, fatigue, or cognitive function^5,33^.

Therefore, the main objective of this systematic review and meta-analysis was to analyze the studies conducted to date, assessing neuropsychological deficits in patients with post-acute COVID-19 syndrome, summarizing the current evidence, and provide a better overview to address this pandemic. Additionally, we assess the quality of the methodology used in each study and propose an outline of the methodology to be followed in future investigations. Furthermore, another objective was to obtain a global index of the magnitude of the effect of post-acute COVID-19 syndrome on the reported cognitive functions.

## Methods

### Search strategy

This work was conducted following the Preferred Reporting Items for Systematic Reviews and Meta-Analyses (PRISMA) guidelines^34^. The protocol was also registered in the Prospective International Registry of Systematic Reviews, PROSPERO (CRD42021260286).

A systematic literature search on PubMed, Cochrane Central Register of Controlled Trials (CENTRAL), Scopus and Web of Science databases was carried out to obtain studies or abstracts published between January 2020 and September 2021. A combined set of keywords has been used to identify human studies that reported on neuropsychological symptoms in patients with persistent COVID symptoms. The keywords entered into the search were as follows: (1) “SARS-CoV-2 OR coronavirus OR COVID-19 OR COVID OR severe acute respiratory syndrome”; (2) “long-COVID OR post-COVID syndrome OR post-acute COVID-19 syndrome OR long-term symptoms”; (3) “neuropsychology OR cognition* OR psychology”; (4) “neuropsychological deficits* OR cognitive deficits* OR cognitive function*” and (5) “Neuropsychological evaluation* OR Neuropsychological assessment* OR “neuropsychological battery* OR cognitive evaluation* OR cognitive assessment* OR cognitive test* OR cognitive screening.” Keywords were combined for a more comprehensive search with OR and AND operators as follows: (1) + (2); (1) + (2) + (3); (1) + (2) + (3); (1) + (2) + (4) and (1) + (2) + (4) + (5) (see supplementary material Table S1 for the search keyword combination details). The search terms were specified to be found in the title and abstract of the studies. In addition, to ensure literature saturation, we searched the references of all identified relevant articles and narrative reviews/systematic reviews and/or meta-analyses to consider their inclusion in this review. After removing duplicates manually and with the help of an automation software^35^, articles were reviewed on the basis of their titles and abstracts separately. Relevant articles were retrieved in full text and subjected to the selection criteria. All the steps mentioned in the search process were conducted independently by two different authors (S.S.-R. and Y.B.-B.). Disagreements were resolved by discussion and, when necessary, by consultation with a third researcher.

### Eligibility criteria

The inclusion criteria established for the studies were: (1) patients with a confirmed diagnosis of SARS-COV-2 infection for at least three weeks before the study; (2) patients with post-acute COVID-19 syndrome undergoing a standardized cognitive function assessment; (3) study design (cohort studies, case–control studies, cross-sectional studies, case series, case reports, or quantitative studies); and (4) preprints and letters only if they described original research containing data on patients with suspected or laboratory-confirmed coronavirus infection. The exclusion criteria were as follows: (1) studies limited to examining cognitive deficits without standardized assessments; (2) studies that investigated indirect effects of coronavirus infections on mental health in uninfected persons mediated by physical distancing measures such as self-isolation or quarantine and without a positive PCR; (3) conference abstracts, as they lacked sufficient information for quality assessment and data extraction, and (4) studies that included a sample with previous pathologies that affect or could affect their cognitive function (e.g. neurodegenerative diseases, neuropsychiatric disorders, acquired brain injury, etc.). Alternatively, to perform the meta-analysis, only the studies that met the following eligibility criteria were selected: (1) to have a healthy control group, and (2) to report a global cognitive score.

### Quality assessment and risk of bias in the included studies

The quality of the studies used in this systematic review was systematically assessed with the Newcastle–Ottawa Scale (NOS) (see Table 1). This scale was developed to assess the quality of non-randomized studies to incorporate quality assessments in the interpretation of meta-analyses of the results obtained^36^. The NOS scale is divided into three dimensions of cross-sectional, or case–control investigations^36^. For each item, the NOS scale has several response options and depending on the answer chosen, it scores (or not) the total evaluation with a star. For example, in the item on the representativeness of cases, the study would score one star if the study sample had a defined time, all cases in a defined catchment area, all cases in a defined hospital or clinic, etc. (response option a.). In this regard, each study assessed can receive a maximum of one star for each numbered item within the categories of *Selection* and *Exposure*. For the *Comparability* category, a maximum of two stars can be awarded depending on the factors controlled for both the case group and the control, obtaining a maximum of 9 points per study for each star obtained^36^. None of the studies analyzed was excluded since the assessed studies had a total quality score above seven points.

**Table 1:** Quality assessment of non-randomized studies using the Newcastle–Ottawa Scale (NOS).

Studies	Selection				Compatibility	Exposure			Overall Quality Score
	Is the case definition adequate?	Representativeness of the cases	Selection of controls	Definition of controls	Comparability of cases and controls based on the design or analysis	Ascertainment of exposure	The same method of ascertainment and controls	Non-response rate	
Del Brutto et al.^43^	a*	a*	a*	a*	**	a*	a*	c	8
Graham et al. ^44^	a*	a*	a*	a*	**	a*	a*	c	8
Miskowiak et al.^45^	a*	a*	a*	a*	a*	a*	a*	c	8
Ortelli et al.^46^	a*	a*	*	*	**	a*	a*	c	8
Triana et al.^47^	a*	a*	*	*	**	a*	a*	c	8
Woo et al.^48^	a*	a*	*	*	**	a*	a*	c	8

A study could receive a maximum of one star (*) per item in the *Selection* and *Exposure* categories, and two stars (**) for *Compatibility*. The asterisk symbol (*) is equivalent to one point.

Once the quality assessment was performed, we used the Risk of Bias In Non-randomized Studies (ROBINS-I) to assess the risk of bias of the different studies selected for the meta-analysis (Fig. S1, see in supplementary material). ROBINS-I tool evaluates the risk of bias of studies that did not use randomization to allocate the subjects, through different domains. Each domain is rated high, low, or unclear, represented by the colors red, green, and yellow, respectively^37^. Two investigators (S.S.-R. and Y.B.-B.) independently evaluated the quality and risk of bias of the studies, obtaining an inter-rater reliability agreement of 81.2%. All disagreements were resolved by discussion and, when necessary, by consultation with a third researcher until consensus was reached.

### Data extraction

Specific information for the systematic review was extracted from the selected studies, which included: (1) author, (2) date of publication, and (3) study design (see Table 2). In addition, the following descriptive variables were extracted from the studies: (4) setting (i.e., country); (5) sample diagnosis (post COVID-19 infection); (6) sample size (N), sex (%) and age (M ± SD); (7) assessment instrument and total score on the assessment scale (M ± SD); and finally, (8) main outcomes.

**Table 2:** Overview of cited studies included that examine neuropsychological symptoms in recovered SARS-COV-2 patients.

Study	Year	Country	Study design	Sample characteristics	Sample size (N), sex (%) and age (M ± SD)	Assessment tool	Results
Alemanno et al.^17^	2021	Italy	Cross-sectional study	COVID-19 patients + 1 month follow-up	N = 87 (71.26% M; 67.23 ± 12.89)	MMSE; MoCA; HRSD	80% had neuropsychological deficits for: visuospatial/executive functions, naming, short- and long-term memory, abstraction and orientation. However, cognitive deficits were associated to the age of the patients. One month follow-up assessments results showed that more than half of the patients still presented deficits, significantly higher than at admission (*p* = .009)
Blazhenets et al.^31^	2021	Germany	Longitudinal study	COVID patients at subacute and chronic stages (six months after onset)	N = 8 (25% W and 75% M; 66 ± 14.23)	MoCA	MoCA performance improved significantly over time (*d* = .97, *p* = .03). However, scores where still bellow the used cut-off value for detection of cognitive impairment (< 26/30). MoCA scores revealed persistent deficits in visuoconstructive and executive functions and especially in memory
Blomberg et al.^55^	2021	Norway	Prospective cohort study	C.P.: Persistent symptoms 6 months after COVID-19 of hospitalized patients (Hosp.) and home isolates (HI) of the first pandemic wave H.C.. = PCR- (seronegative household contacts)	H.C. = 60 (63%M; Median = 29 (14–48)) C.P = 312 (HI = 247; Hosp. = 65) M = 51%; Median = 46 (30–58)	Memory-concentration test	61% (189/312) of the total patient population had persistent symptoms 6 months after the initial COVID-19 illness, with the most common symptoms being fatigue (37%), difficulty focusing (26%), altered smell and/or taste (25%), memory problems (24%), and dyspnea (21%)
Del Brutto et al.^43^	2021	Ecuador	Prospective, cross-sectional study	C.P = PCR + H.C. = PCR-(All participants were from the Atahualpa Project, and had a similar socioeconomic status and lifestyles)	C.P = 52 (62%W and 38%M; 59.4 ± 10.6) H.C. = 41 (66%W and 34%M; 66.6 ± 10.6)	MoCA	The post-COVID MoCA scores were significantly lower than pre-pandemic mean scores among seropositive individuals (21.7 ± 4 vs. 19.6 ± 4.2;* p* = .010) but not in their seronegative counterparts (21.5 ± 5 vs. 21 ± 4; *p* = .618)
Ferrucci et al.^24^	2021	Italy	Prospective, cross-sectional study	COVID recovered patients (previously hospitalized)	N = 38 (29% W and 71% M; 53.45 ± 12.64)	MoCA, BRB-NT	Five months after hospital discharge, 60.5% had cognitive abnormalities: 42% showed decreased cognitive processing speed and about 20% had verbal and spatial long-term memory dysfunction
Frontera et al.^25^	2021	USA	Prospective longitudinal study	Patients recovered from COVID with neurological complications (NC); and ones with no NC (nNC) (All of them were previously hospitalized)	NC = 196 nCN = 186	MoCA, Neuro-QoL	Fifty percent of the patients presented cognitive deficits with no significant differences between the two groups
Graham et al. ^44^	2021	USA	Prospective study	C.P.: Non-hospitalized patients recovered from COVID H.C..: Could have been exposed to the virus but had a PRC-	H.C. (PCR-) = 12 (26%M and 74%W; 42.6 ± 10.8) C.P (PCR +) = 22 (34%H and 66%M; 43.7 ± 11.8)	NIH Toolbox	The COVID patients performed worse on cognitive tasks of attention and working memory compared to a demographically matched U.S. population
Groiss et al.^21^	2020	France	Case series	Patients recovered from severe COVID-19 (required mechanical ventilation)	N = 4 (100% M, 59.5 ± 17.6)	MoCA, SDMT, MME	All patients showed clinically relevant cognitive impairment. Fifty percent of patients had moderate MoCA cognitive impairment
Hampshire et al.^56^	2021	United Kingdom	Longitudinal study (online)	Recovered COVID patients with post-COVID-19 symptoms (different levels of virus severity reported)	N = 81,337 (55%W; 46.7 ± 15.7)	Digit Span; Rare word definitions; Analogical reasoning; Target Detection; 2D mental rotations; Spatial span; Block rearrange; TofL; Face emotional discrimination	Deficits had a significant effect size for individuals who had been hospitalized
Hellmuth et al.^54^	2021	USA	Two-case study	COVID recovered patients (Non-hospitalized)	N = 2 (100% W; 44.5)	MoCA	Results revealed impairments in working memory and digit span backwards, with high average attention skills several days after COVID recovery (at least 72 days). Other cognitive domains appeared normal
Henneghan, et al.^66^	2021	USA	Observational, cross-sectional analysis	Symptoms four months after the illness. Most had a history of mild or moderate COVID-19 severity	C.G = 52 (78.85% W; 37.33 ± 12.12 )	BrainCheck Memory battery, The Patient-Reported Outcome Measures Information System (PROMIS) Item Bank v2.0 Cognitive Function Short Form 8a (PROMIS Cognitive)	40% of the participants scored more than one standard deviation below the "normal" population mean on one or more of the cognitive tests. The results, therefore, indicate the presence of deficits in cognitive functioning
Hosp et al.^32^	2021	Germany	Prospective cohort study	COVID recovered patients	N = 29 (62%W and 38%M; 65.2 ± 14.4)	MoCA	MoCA domain scores revealed particular impairment in the domains of executive skills, visual construction, memory, and attention. Language and orientation were unaffected
Jaywant et al.^19^	2021	USA	Cross-sectional study	Patients recovered from prolonged hospitalization for COVID 19 who required acute inpatient rehabilitation before discharge (43.2 ± 19.2) days after initial admission	N = 57 (75%M; 64.5 ± 13.9)	Brief Memory and Executive Test (BMET)	Deficits were common in working memory (26/47 [55%] of patients), set-shifting (21/44 [47%]), divided attention (18/39 [46%]), and processing speed (14/35 [40%]). Executive dysfunction was not significantly associated with the duration of intubation or time from extubation to assessment, psychiatric diagnosis, or preexisting cardiovascular/metabolic disease. Attention and executive functions are frequently impaired in COVID-19 patients requiring acute rehabilitation before discharge
Leth et al.^57^	2021	Denmark	Longitudinal study	Six- to 12-week follow-up of hospitalized COVID-19 patients	N = 49 (57% W; 58)	OMC test	The main persistent symptoms at 6 and 12 weeks of follow-up were fatigue, dyspnea, altered concentration, cough, and altered smell and taste, in agreement with the literature so far
Miskowiak et al.^45^	2021	Denmark	Prospective study	C.P = COVID recovered patients (previously hospitalized) H.C. = healthy controls with no previous exposure to the virus	H.C = 100 (59% W y 41% M; 56 ± 6.9) C.P = 29 (41%W and 59% M; 56.2 ± 10.6)	SCIP-D, TMT-B	Moderate impairments were observed in working memory, verbal fluency and psychomotor speed. Compared to H.C.., C.P. showed global cognitive impairments with moderate to large effect size (total SCIP: *t* = −2.78, *gl* = 35.3, *p* = 0.01; Cohen's *d* = −0.70) and moderate impairments in verbal learning and working memory (VLT-1: *t* = −3. 06, gl = 127, *p* = 0.003, Cohen's *d* = −0.62; WMT: *t* = −2.11, *gl* = 34.0, *p* = 0.04, Cohen's *d* = −0.44)
Ortelli et al.^46^	2021	Italy	Prospective study	C.P: COVID recovered patients (hyperinflamatory state during the acute phase) H.C.: age-and sex-matched healthy individuals	H.C. = 12 (33.3% W and 66.7% M; 64.3 ± 10.5) C.P = 12 (16%W and 74% M; 67 ± 9.6)	MoCA & FAB	MoCA revealed significantly poorer performance in patients compared to H.C (*p* < .001), particularly in the executive domain
Patel et al.^58^	2021	USA	Cross-sectional observational study	COVID recovered patients	N = 77 (63.6% M and 36.4% W; 61.03 ± 15.67)	MoCA	80.5% of patients demonstrated cognitive deficits on MoCA at admission: 50.6% were mildly impaired, 26% moderately impaired, and 3.9% severely impaired. Cognitive impairment was not associated with age or duration of acute care hospitalization. 71.1% of patients with discharge scores improved and reached the minimum clinically important MoCA difference; however, 77.8% continued to score in the impaired range
Pilotto et al.^67^	2021	Italy	Prospective study	COVID-19 recovered patients	N = 165 (30.3% W; 64.8 ± 12.6)	MoCA	Cognitive deficits primarily in memory and attention
Pinnock et al.^68^	2021	Canada	Prospective cohort study	Post-COVID-19 syndrome in patients 1.5 years after discharge	N = 28 (93% W; 46.7 ± 10)	WMS-III Orientation subtest; WASI (Matrix Reasoning; Vocabulary) DKEFS Trail Making Test; DKEFS Stroop; TEA Lottery subtest; WMS-III (Digit Span; Spatial Span; Logical Memory; Faces subtest); Consonant Trigrams; Ruff 2 & 7 Selective Attention Test; PASAT; BNT (60-item); D-KEFS Verbal Fluency; RCFT; CVLT-II; Event and Time-Based Prospective Memory; MAC-S Revised; Beck Depression Inventory (BDI-II); Beck Anxiety Inventory (BAI)	Individuals who recovered from the disease showed chronic cognitive difficulties 1.5 years after the disease on complex attention and working memory tasks that rely on executive thinking skills. In addition, participants who received oxygen performed poorly on most attention-shifting tasks, although their overall performance on these tasks was within normal limits Approximately half of the participants exhibited slower processing speed when performing tasks requiring controlled attention and concentration, suggesting increased mental effort. Participants performed within normal limits on simple measures of attention and concentration, immediate and delayed memory, reasoning skills, visuospatial ability, and language. Participants also endorsed poorer memory ability than their age-matched peers and mild to moderate symptoms of depression and anxiety
Ramani, et al.^69^	2021	USA	Prospective study	COVID-19 recovered patients	N = 28 (39.3% W and 60.7% M; 55.5 ± 11.9)	MoCA	None of the patients had clinically diagnosed depression, cognitive impairment or insomnia prior to admission. During follow-up. Assessment of mild cognitive impairment was more frequent according to MoCA (57.14%)
Triana, et al.^47^	2020	Cuba	Prospective study	C.P = COVID-19 recovered patients H.C. = healthy participants with with no exposure to any relevant disease in the previous month and a negative PCR	H.C. = 100 (56% W and 44% M; 50.45 ± 12.58) C.P = 42 (52.4% W and 47.6% M; 54.55 ± 12.5)	MoCA	Inferior performance of patients was found in the variables: working memory (*p* = .005), attention (*p* = .026), abstraction (*p* = .021), delayed memory (*p* = .001) and in the MoCA total score (p = .007). Significant correlations were found between MoCA score and age (*r* = -.520, *p* = .001), educational level (*r* = .551, *p* = .000), forced vital capacity (FVC) [(*r* = .667, *p* = .000)], forced expiratory volume (FEV1)[(*r* = .573 (*p* = .001)] and 6-min walk test (6MWD) [(r = .563, *p* = .002)]. Predictors of cognitive performance were FVC, 6MWD and schooling
Venturelli et al.^70^	2021	Italy	Prospective cohort study	Hospitalized patients with COVID-19	N = 767 (32.9% W; 63 ± 13.6) (only 304 patients did the MoCA test)	MoCA	Only two of the 304 patients who were evaluated with the MoCA obtained pathological scores, although 69 reported related symptoms
Voruz et al.^26^	2021	Switzerland	Prospective and cross-sectional study	COVID-19 recovered patients	N = 45 (55 ± 2)	WAIS-IV, MEM III, ROCF, Stroop test, TMT, TAP, VOSP	Cognitive deficits common to all three groups were observed in long-term episodic memory, the verbal and visual domains, executive functions (e.g., inhibition and mental flexibility, and both categorical and literal verbal fluency), sustained and divided attention, and language (semantic matching and naming)
Woo, et al.^48^	2020	Germany	Cross-sectional study	C.P.: COVID-19 recovered patients (only those who did not stay in the intensive care unit) H.C.: healthy employees of the medical centre, with a negative PCR	H.C. = 10 (40% W and 60% M; 38.4 ± 14.4) C.P = 18 (57.9% W and 42.1% M; 42.2 ± 14.3)	TICS-M (PT/50)	Post-COVID-19 patients scored significantly lower on the TICS-M (x = 38.83; range, 31–46) compared to healthy controls (x = 45.8; range, 43–50). 50% reported attention deficits, 44.4% concentration deficits, 44.4% short-term memory deficits, 27.8% word-finding difficulty, and 16.7% fatigue
Zhou et al.^33^	2020	China	Prospective cohort study	Post-COVID-19 syndrome patients	H.C. = 29 (42.48 ± 6.94; 41.37%M) C.P = 29 (47 ± 10.54; 62%M)	TMT (Trail Making Test); SCT; CPT	COVID-19 patients showed lower performance in CPT compared to the control group. In addition, there was a trend of significant difference in the reaction times of CPT 1 and CPT 2 and the correct number of CPT 2 between the groups. However, there was no significant difference between the two groups in TMT, SCT or DST performance

*H.C.* healthy control group, *C.P* COVID-19 patients, *PCR* polymerase chain reaction test, *W* women, *M* men, *HSRD* Hamilton Rating Scale for Depression, *WAIS-IV* Wechsler Memory Scale-Third Edition, *ROCF* Rey–Osterrieth complex figure test, *TMT* trail making test, *TAP* test for attentional performance, *BRNT-NT* brief repeatable battery of neuropsychological tests, *Neuro-QoL* quality of life in neurological disorders, *SDMT* symbol digit modality test, *FAB* frontal assessment battery, *VOSP* visual object and space perception battery, *TofL* tower of London, *TICS-M* the modified telephone interview for cognitive status, *SCIP-D* screen for cognitive impairment in psychiatry danish version, *STC* sign coding test, *CPT* continuous performance test, *BNT* Boston naming test, *OMC* orientation, memory and concentration test.

In the meta-analysis, all effect sizes were calculated from the means and standard deviations. Effect sizes for general cognitive functions were estimated based on the mean scores (with 95% CI) extracted from cognitive screening tests. This was, specifically, the means corresponding to the control group (healthy individuals) and the experimental group (patients with persistent COVID-19 symptoms). For this purpose and due to the variability of the neuropsychological tests used by the studies, those that provided information with different cognitive screening tools, such as MoCA, NIH Toolbox, SCIP or TICS-M, were included in the analyses. The same measures were selected for the sensitivity analyses.

### Statistical analysis

A meta-analysis was conducted to compare the means of cognitive deficits in recovered COVID patients and healthy control participants. We estimated the standardized mean difference (SMD) statistic with a 95% confidence interval (CI) from the means and standard deviations (SD) using Cohens’*d* formula^38^. All of them were later corrected by Hedges’ *g* sample size bias adjustment formula^39,40^. For the main analyses, we used the random-effects model because of the expected high heterogeneity, and pooled results of means and their respective 95% CIs were estimated using the Inverse method of variance.

The *I*^*2*^ index was used to quantify statistical heterogeneity among the selected studies. The heterogeneity test measures the degree of inconsistency among the results of studies and is interpreted as the approximate proportion of the total variation in study estimates due to heterogeneity rather than sampling error^41^. Heterogeneity (*I*^2^) was classified as low (below 25%), moderate (25–75%), and high (above 75%)^41^. We did not conduct the funnel plot for asymmetry due to an insufficient number of studies (n < 10).

Sensitivity analyses were performed to assess the contribution of individual studies to the overall results of the meta-analysis. All data were analyzed with the Review Manager Software^42^ (RevMan 5, Version 5.4.1); with the significance threshold set at *p* values less than 0.05.

## Results

### Study selection

**Figure 1:**
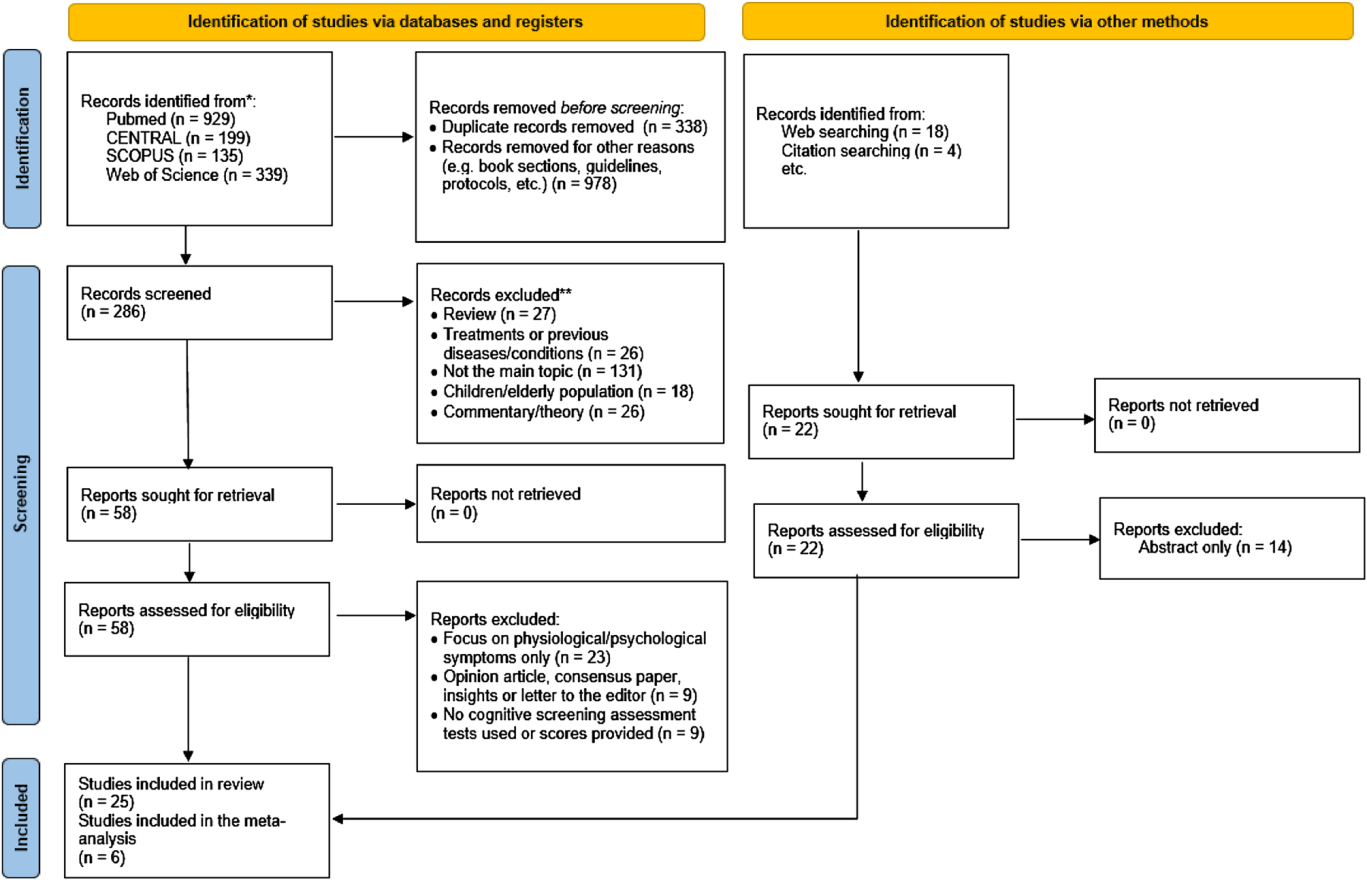
PRISMA summary of articles included in this meta-analysis. * eligibility criteria for meta-analysis was followed the eligibility criteria for meta-analysis studies, previously mentioned in methods, have been applied.

Initially, 1602 studies were identified in PubMed, Cochrane Central Register of Controlled Trials (CENTRAL), Scopus and Web of Science databases, and 22 additional studies were identified through other sources (citation searching). Three hundred thirty-eight articles were eliminated because they were duplicates (267 of them were detected by EndNote reference manager software). From the remaining 1286 articles, 978 were also discarded because of other reasons (e.g. book sections, protocols, guidelines, etc.). After that, 308 studies remained, which were reduced to 80 articles by reading titles and abstracts. Therefore, 80 papers were fully read and assessed for eligibility. Of those studies, 32 did not meet the previously established eligibility criteria, 14 had insufficient data, and nine were opinion articles, consensus papers o letters to the editor. Finally, 25 studies were selected for the systematic review. Lastly, for the meta-analysis, of the 25 studies included in the systematic review, only 6 of them met the eligibility criteria (having a control group and reporting an overall cognitive score) for conducting the quantitative analyses (see Fig. 1).

### Characteristics of the studies

Six studies, including 175 patients with persistent COVID-19 symptoms and 275 healthy persons met the above inclusion criteria and were included in the meta-analysis^43–48^. The selected studies were published between December 2020 and April 2021, and the number of participants involved in the studies ranged from 10 to 100. The control group (healthy individuals) of the studies consisted of 150 women (54.72%) and 125 men (45.28%) with a mean age of 53.06 ± 10.96 years old. Moreover, the experimental group (patients with persistent COVID symptoms) was composed of 89 women (50.88%) and 86 men (49.12%) with a mean age of 53.84 ± 11.57 years.

### Cognitive dysfunction in patients with post-acute COVID-19 syndrome

**Figure 2:**
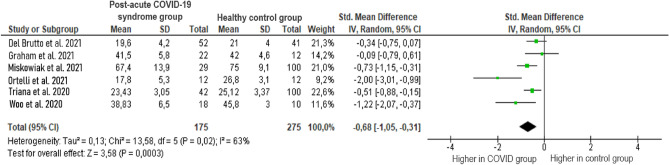
Comparison of cognitive impairments reported by studies in patients with post-acute COVID-19 syndrome and healthy volunteers.

The effect size of overall cognitive functions was based on the mean overall cognitive status of the post-acute COVID-19 syndrome patient group and the healthy control group. The results showed a medium–high overall effect size (*g* =−0.68, *p* = 0.02) with a 95% CI (−1.05 to −0.31). The heterogeneity test showed a significantly moderate level of heterogeneity between studies (*Z* = 3.58, *p* < 0.001; *I*^2^ = 63%). The results of the analysis are displayed in Fig. 2. These results indicate that cognitive dysfunctions are more common in patients with persistent COVID symptoms than in healthy volunteers.

### Sensitivity analysis

**Figure 3:**
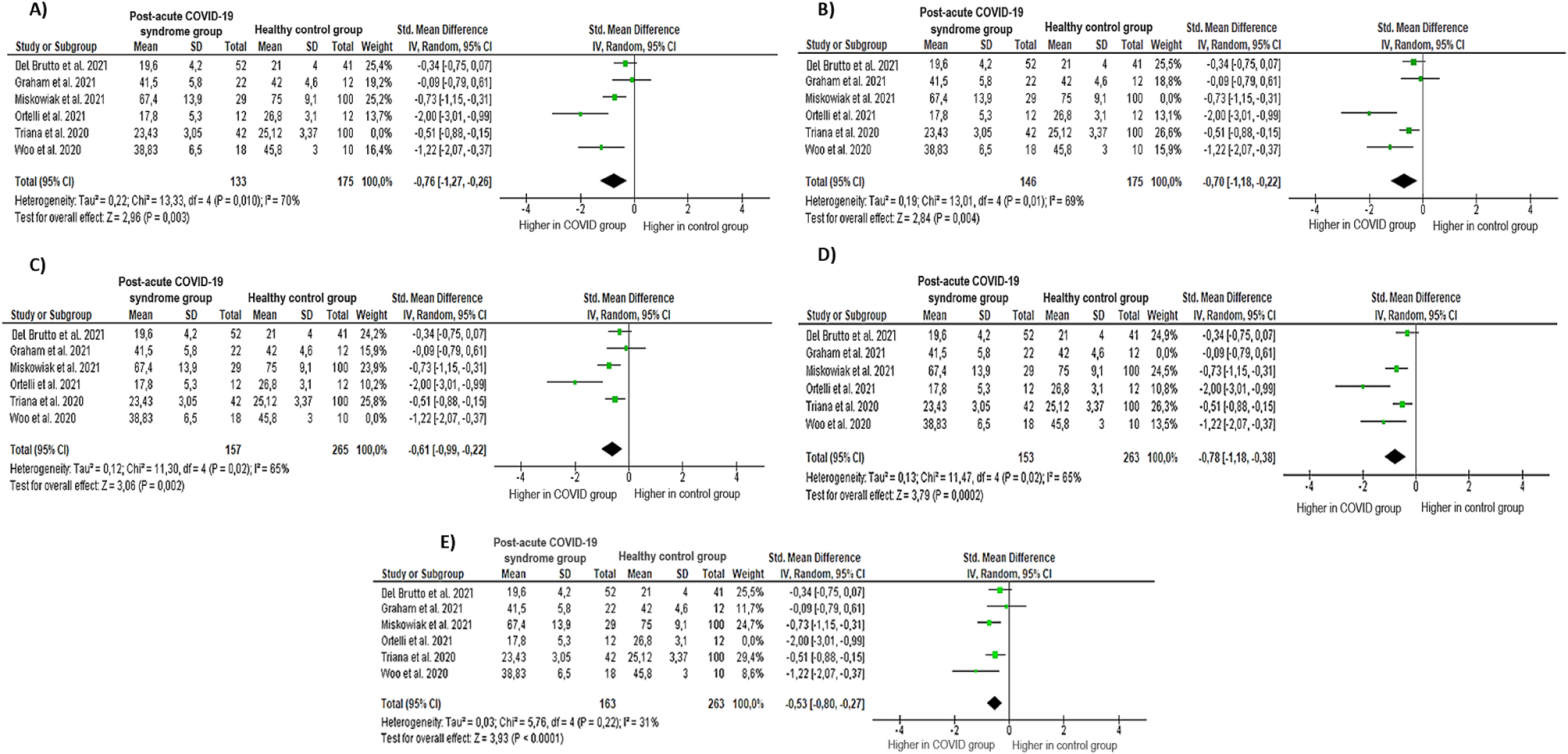
Sensitivity analysis. Note: (**A**) Results without Triana et al. study; (**B**) results without Miskowiak et al. study; (**C**) results without Woo et al. study; (**D**) results without Graham et al. study; and (**E**) results without Ortelli et al. study.

Furthermore, due to the moderate heterogeneity of the studies, we performed a sensitivity analysis to inspect the influence of each study individually for the overall results of the meta-analysis described above (see Fig. 3). First, sensitivity analyses were conducted to examine whether the previously obtained results would change if the study by Triana et al.^47^ was excluded from the analyses because it had the greatest weight of the included studies. Secondly, sensitivity analyses were performed excluding also the study by the authors of Del Brutto et al.^43^, and so on until it was verified that deleting all the articles did not change the heterogeneity to any significant extent. Despite this, the exclusion of the studies did not significantly change the size or significance of the above domains. The exception was observed in the sensitivity analysis in which the study by Ortelli et al.^46^ was deleted; heterogeneity dropped to 31% (*Z* = 3.93, *p* = 0.22; *I*^2^ = 31%). Figure 3 shows all the sensitivity analysis results.

## Discussion

The present study provided a comprehensive review examining the link between post-acute COVID-19 syndrome and neuropsychological manifestations after infection recovery. Twenty-five different studies were identified and reviewed that reported data on the post-disease neuropsychological characteristics of COVID-19 infection, of which six were analyzed in this meta-analysis^43,44,46–48^.

The main findings of this systematic review and meta-analysis showed an increased likelihood of exhibiting cognitive deficits in patients who recovered from COVID-19^43,44,46–48^. Furthermore, the evidence indicated that the prevalence of cognitive deficits was high in patients who, after recovery from COVID, continued to suffer from disease symptoms. However, it should be noted that the lack of a standard protocol for baseline cognitive assessment of patients' cognitive performance may complicate the comparison of the results obtained by different studies. Also, failure to report or assess psychological (e.g., stress, anxiety, etc.), socioeconomic (e.g., unemployment, few resources, lack of support networks, etc.), and contextual (e.g., specific COVID-19 measures such as social distancing) factors possibly affected by the pandemic may complicate the distinction between the effects on cognition of infection and the overall impact of the pandemic. Furthermore, another important variable that has not considered is the role of cognitive reserve (CR). CR is a protective factor against possible neuropsychological deficits^49^, in addition to being associated with neurobiological structures (brain reserve)^50^ that could make individuals less susceptible to brain alterations caused by the virus. On the other hand, stress or anxiety disorders, among others, can affect cognitive functioning^51,52^, and it has been reported that psychological problems have increased since the pandemic^53^. It would therefore be advisable to report such data for a better understanding of the consequences of the infection. Regarding severity, mean scores of cognitive deficits were below clinical cut-offs^43,44,46–48^. Nevertheless, studies such as Alemanno et al.^17^ concluded that scores on assessment scales improved relative to patients with the virus at the time of assessment, although cognitive deficits in patients with persistent COVID were lower than in healthy controls.

The main results obtained from the meta-analysis showed moderate differences in global cognitive status (*g* = −0.70) between recovered coronavirus patients with persistent symptomatology and healthy controls. These results support the information reported by other studies that conducted follow-ups on patients with persistent COVID symptoms. They found that approximately 30–80% of these patients developed long-term COVID symptoms lasting one to 6 months, with the most frequent symptoms being fatigue and neuropsychological deficits in executive functions^26,31,32^; working memory^24,44,47,54^; processing speed^19,21,24^; and attention^19,31,32,44,47,48^.

On the other hand, it should be noted that the scientific literature reviewed in this study reported data from both hospitalized and non-hospitalized patients (i.e., home isolation) depending on the studies. Therefore, it is important to highlight that the patients evaluated in the different studies analysed experienced different levels of severity of the disease in the acute phase. Some studies evaluate patients with mild COVID-19 symptoms that mostly did not require hospitalization or the intensive care unit^43,44,48^, while others evaluate patients with more severe COVID-19 symptoms that required hospitalization^45,46^. In particular, a worse cognitive performance could be observed in hospitalized patients compared to non-hospitalized patients^19,24,45,55–58^. This could be due to a more severe alteration of brain structures vulnerable to COVID-19^59^, and as a consequence a worse cognitive performance. Therefore, when interpreting the results obtained in this meta-analysis, it is necessary to consider the variability in the cognitive performance of individuals.

Moreover, not all studies report extensive details of the characteristics of the matched healthy controls. And among the studies that do so, differences can be observed among the healthy participants: in some studies, the healthy participants have lived with the virus at home but have not been infected^55^, others have not been previously exposed to the virus^43,45,47^ and others can not to ensure it^44,48^. Therefore, caution must be taken when interpreting the results and generalizing the findings regarding the long-term side effects of COVID-19, especially for all patients who did not require hospital admission and therefore underwent different levels of symptomatology severity.

The information available so far suggests that, in the acute stage of the infection, cognitive deficits are a common feature^17–19,22^, so it is probably considered a major clinical problem. However, conclusions should be made with caution since the information available on the acute consequences of pathology is limited and there is no specific data on the pathogenesis of the post-acute phase caused by the virus. The etiology of the neuropsychological consequences of coronavirus could be multifactorial. Several causes could affect cognition, such as the direct effects of the virus, the extent of symptoms such as hypoxia, cerebrovascular disease, the immunological response, medical resources and treatments, social isolation and psychological impact of the pandemic, or the concern of infecting others^21,26^. There is also a link between inflammation and cognitive dysfunction that could explain some of the neuropsychological morbidity^16,60–62^.

In line with the results obtained in the studies included in this review and meta-analysis, the research by Rogers and colleagues^16^ concluded that, following recovery from pre-COVID-19 coronavirus infections (e.g., MERS and SARS) and during a follow-up period of between six weeks and 39 months, sleep disturbance, emotional lability, reduced concentration levels, memory deficits, and fatigue were reported in more than 15% of patients^16^. Therefore, further research and monitoring of the health consequences of the current pandemic are needed.

### Limitations and future studies

This study has several limitations that offer opportunities for future research. The main limitations of this meta-analysis include the use of non-peer-reviewed preprint papers, the exclusion of manuscripts published in a language other than English or Spanish, as well as the selection of studies with small sample sizes. In addition, few studies included healthy comparison groups, since a large majority compared the results of patients with post-acute COVID-19 syndrome with the standardized mean score of the test itself in the general population. Another significant limitation that may have affected the research conducted has been the mobility restriction measures applied worldwide, which have reduced the possibilities for data collection to online and/or remote formats (e.g., telephone assessment, online questionnaires, etc.). At the same time, the need, timing and process of publishing information about COVID-19 to facilitate the development of guidelines for clinicians may have affected the methodological quality of some studies. Jung et al.^63^ assessed the methodological quality of 686 COVID-19 articles and compared them with control articles published before the pandemic. The authors observed that COVID-19 articles were associated with lower methodological quality scores when published within a short time period^63^.

Another limitation was that there was considerable variation in the follow-up period (between approximately 1 and 5 months) in the studies that evaluated the long-term impact of the virus, which made comparability difficult. These may have also contributed to the heterogeneity present in this study. In addition, different studies used tests such as the Montreal Cognitive Assessment Test (MoCA) to assess cognitive impairment in these patients. The test is a cognitive screening scale validated in different populations and ages but mainly used in people older than 50 years and in the context of possible dementia. Although its purpose is to detect cognitive impairment, more sensitive neuropsychological tests such as the Mini-Mental State Examination (MMSE) (which has been shown to have a high sensitivity in neurocognitive diseases) would be needed^64,65^. Related to this, another limitation of the present study is to have placed the focus of analysis on global cognitive function, rather than on specific cognitive domains. Although this decision was due to a lack of resources (e.g., insufficient studies when the review was initiated), the analysis of data from extensive (domain-specific) neuropsychological assessments is recommended for future research in order to obtain more detailed data. Finally, although statistical methods were used to standardize the scores of all the studies, the different scales used by the included studies may have also contributed to the heterogeneity shown in this meta-analysis.

Future prospective studies on the subject are urgently needed. It is highly encourage that future studies systematically evaluate the prevalence of neuropsychological symptoms in patients with post-acute COVID-19 syndrome. It would be ideal to assess mental health prior to infection; as well as other relevant factors such as cognitive reserve, lifestyle or socioeconomic status, among others. In addition, it would be interesting if most of the studies used a common or similar, comprehensive psychological assessment test that could detect more specific cognitive deficits.

The long-term effect of the coronavirus infection on quality of life and eventual return to normalcy due to productivity loss and persistent cognitive impairment may be significant as the pandemic continues to escalate. Future longitudinal studies are needed to further investigate the cognitive impact of the infection on non-hospitalized people, as they constitute the majority of patients with COVID-19 and may have a significant impact on workforce productivity.

### Conclusion and clinical implications

The main objective of this study was to shed light on a scarcely examined issue and to provide some structure to the growing evidence supporting the importance of assessing cognitive deficits in patients with persistent COVID symptoms^19,31,32,44,47,48^.

COVID-19 disease is a serious and increasingly widespread social and mental health problem and is becoming the focus of empirical research by investigators worldwide. The current findings support previous results by demonstrating significant relationships between persistent COVID symptoms and neuropsychological deficits. Importantly, the findings of the current study extend those of previous research by providing initial evidence that COVID-19 may affect the CNS increasing the likelihood of neuropsychological deficits even weeks after the infection. In summary, the current study makes an important contribution to understanding the relationship between post-acute COVID-19 syndrome and neuropsychological deficits.

Furthermore, it is very important to take into account the strong relationship between neurological and psychiatric symptoms and cognitive deficits in the study of COVID-19 and its consequences. This relationship is crucial to consideration when treating the disease and subsequent rehabilitation of COVID-19 patients, especially in the clinical setting. Therefore, it is important to take these factors into account for future studies and their impact on clinical practice.

While these findings may provide relevant information for the prevention and treatment of such cognitive dysfunctions, additional research is required to further investigate and define the possible mechanisms that could produce the virus-related cognitive alterations.

In conclusion, although there are multiple potential ways in which this pandemic could affect mental health, the present review suggests, firstly, that a high percentage of recovered COVID-19 individuals suffer from neuropsychological deficits in the aftermath of coronavirus infection and, secondly, that there are few existing studies to suggest that common neuropsychological difficulties are a feature of post-acute COVID-19 syndrome. Nevertheless, the present systematic review emphasizes that professionals treating patients with persistent COVID symptoms should be aware of this phenomenon and incorporate standard tests assessing cognitive function (e.g., MoCA) in their routine evaluation, to avoid the adverse consequences already reported in several studies. Furthermore, collecting all the results obtained in the different investigations reviewed in this study, months after hospital discharge, increased fatigue and problems in concentration, memory and cognitive speed are reported, which could disrupt daily life. Therefore, patients and essential workers could benefit from early neuropsychological testing to evaluate the impairment degree after COVID-19 hospitalization and its potential impact on their return to everyday activities. Future studies should carefully assess the long-term overall course of cognitive deficits in patients with post-acute COVID-19 syndrome as well as the effectiveness of rehabilitation treatments, particularly in younger patients. As research on neuropsychological symptoms in patients with post-acute COVID-19 syndrome advances, the need to examine this symptomatology in a multidisciplinary manner to achieve greater scientific evidence to corroborate the incidence and prevalence of COVID is increasingly clear.

## Supplementary Information

Supplementary Information.

## Data Availability

The datasets used and/or analysed during the current study available from the corresponding author on reasonable request.

## References

[1] World Health Organization. Global COVID-19 Clinical Platform Case Report Form (CRF) for Post COVID condition (Post COVID-19 CRF). (2021).

[2] Chen, T. *et al.* Clinical characteristics of 113 deceased patients with coronavirus disease 2019: Retrospective study. *BMJ* **368**, (2020). 10.1136/bmj.m1091 PMC7190011 32217556

[3] Wang X (2020). Clinical characteristics of non-critically ill patients with novel coronavirus infection (COVID-19) in a Fangcang Hospital. Clin. Microbiol. Infect..

[4] Haidar MA (2020). Neurological and neuropsychological changes associated with SARS-CoV-2 infection: New observations, new mechanisms. Neuroscientist.

[5] Helms J (2020). Neurologic features in severe SARS-CoV-2 infection. N. Engl. J. Med..

[6] Mao L (2020). Neurological manifestations of hospitalized patients with COVID-19 in Wuhan, China: A retrospective case series study. SSRN Electron. J..

[7] Chen X (2021). A systematic review of neurological symptoms and complications of COVID-19. J Neurol.

[8] Asadi-Pooya AA, Simani L (2020). Central nervous system manifestations of COVID-19: A systematic review. J Neurol Sci.

[9] Divani, A. A. *et al.* Central nervous system manifestations associated with COVID-19. *Curr Neurol Neurosci Rep.* **20**, (2020). 10.1007/s11910-020-01079-7 PMC7599061 33128130

[10] Klironomos S (2020). Nervous system involvement in coronavirus disease 2019: Results from a retrospective consecutive neuroimaging cohort. Radiology.

[11] Speth MM (2020). Mood, anxiety and olfactory dysfunction in COVID-19: Evidence of central nervous system involvement?. Laryngoscope.

[12] Beach SR (2020). Delirium in COVID-19: A case series and exploration of potential mechanisms for central nervous system involvement. Gen Hosp Psychiatry.

[13] Liguori C (2021). Depressive and anxiety symptoms in patients with SARS-CoV2 infection. J Affect Disord.

[14] Nalleballe K (2020). Spectrum of neuropsychiatric manifestations in COVID-19. Brain Behav Immun.

[15] Varatharaj A (2020). Neurological and neuropsychiatric complications of COVID-19 in 153 patients: a UK-wide surveillance study. Lancet Psychiatry.

[16] Rogers JP (2020). Psychiatric and neuropsychiatric presentations associated with severe coronavirus infections: a systematic review and meta-analysis with comparison to the COVID-19 pandemic. Lancet Psychiatry.

[17] Alemanno F (2021). COVID-19 cognitive deficits after respiratory assistance in the subacute phase: A COVID rehabilitation unit experience. PLoS ONE.

[18] Beaud V (2021). Pattern of cognitive deficits in severe COVID-19. J Neurol Neurosurg Psychiatry.

[19] Jaywant A (2021). Frequency and profile of objective cognitive deficits in hospitalized patients recovering from COVID-19. Neuropsychopharmacology.

[20] Whiteside DM (2021). Neurocognitive deficits in severe COVID-19 infection: Case series and proposed model. Clin. Neuropsychol..

[21] Groiss SJ (2020). Prolonged neuropsychological deficits, central nervous system involvement, and brain stem affection after COVID-19: A case series. Front Neurol.

[22] Amalakanti S, Arepalli KVR, Jillella JP (2021). Cognitive assessment in asymptomatic COVID-19 subjects. Virusdisease.

[23] Iadecola C, Anrather J, Kamel H (2020). Effects of COVID-19 on the nervous system. Cell.

[24] Ferrucci R (2021). Long-lasting cognitive abnormalities after COVID-19. Brain Sci.

[25] Frontera, J. A., Yang, D., Lewis, A. & Patel, P. A prospective study of long-term outcomes among hospitalized COVID-19. *medRxiv* (2021). 10.1016/j.jns.2021.117486 PMC8113108 34000678

[26] Voruz, P. *et al.* Long COVID neuropsychological deficits after severe, moderate or mild infection. *medRxiv* 2021.02.24.21252329 (2021) 10.1101/2021.02.24.21252329.

[27] Huang C (2021). 6-month consequences of COVID-19 in patients discharged from hospital: a cohort study. Lancet.

[28] Maltezou HC, Pavli A, Tsakris A (2021). Post-COVID syndrome: An insight on its pathogenesis. Vaccines (Basel).

[29] Nalbandian A (2021). Post-acute COVID-19 syndrome. Nat Med.

[30] World Health Organization. A clinical case definition of post COVID-19 condition by a Delphi consensus,. https://www.who.int/publications/i/item/WHO-2019-nCoV-Post_COVID-19_condition-Clinical_case_definition-2021.1 (2021).

[31] Blazhenets, G. *et al.* Slow but evident recovery from neocortical dysfunction and cognitive impairment in a series of chronic COVID-19 patients. *J. Nucl. Med.* 121.262128 (2021) 10.2967/jnumed.121.262128. 10.2967/jnumed.121.262128 PMC8882885 33789937

[32] Hosp, J. A. *et al.* Cognitive impairment and altered cerebral glucose metabolism in the subacute stage of COVID-19. **144**, 1263–1276 (2021). 10.1093/brain/awab009 PMC8083602 33822001

[33] Zhou H (2020). The landscape of cognitive function in recovered COVID-19 patients. J Psychiatr Res.

[34] Page, M. J. *et al.* PRISMA 2020 explanation and elaboration: Updated guidance and exemplars for reporting systematic reviews. *BMJ* vol. 372. 10.1136/bmj.n160 (2021). 10.1136/bmj.n160 PMC8005925 33781993

[35] The EndNote Team. EndNote. Preprint at (2013).

[36] Wells, G. *et al.* The Newcastle-Ottawa Scale (NOS) for assessing the quality of nonrandomised studies in meta-analyses (2000).

[37] Sterne JA (2016). ROBINS-I: A tool for assessing risk of bias in non-randomised studies of interventions. BMJ (Online).

[38] Cohen, J. *Statistical Power Analysis for the Behavioral Sciences*. (Routledge Academic, 1988).

[39] Cumming, G. *Understanding the New Statistics: Effect sizes, Confidence Intervals, and Meta-Analysis.* (Routledge, 2012).

[40] Lakens D (2013). Calculating and reporting effect sizes to facilitate cumulative science: A practical primer for t-tests and ANOVAs. Front Psychol.

[41] Higgins JPT, Thompson SG (2002). Quantifying heterogeneity in a meta-analysis. Stat Med.

[42] Review Manager 5 (RevMan).

[43] Del Brutto, O. H. *et al.* Cognitive decline among individuals with history of mild symptomatic SARS-CoV-2 infection: A longitudinal prospective study nested to a population cohort. *Eur. J. Neurol.* 1–9 (2021) doi:10.1111/ene.14775. 10.1111/ene.14775 PMC8014083 33576150

[44] Graham EL (2021). Persistent neurologic symptoms and cognitive dysfunction in non-hospitalized Covid-19 “long haulers”. Ann Clin Transl Neurol.

[45] Miskowiak, K. W. *et al.* Cognitive impairments four months after COVID-19 hospital discharge: Pattern, severity and association with illness variables. *Eur. Neuropsychopharmacol.* 39–48 (2021) 10.1016/j.jns.2020.117271. 10.1016/j.euroneuro.2021.03.019 PMC8006192 33823427

[46] Ortelli P (2021). Neuropsychological and neurophysiological correlates of fatigue in post-acute patients with neurological manifestations of COVID-19: Insights into a challenging symptom. J Neurol Sci.

[47] Triana RM (2020). Cognitive performance in convalescent covid-19 patients. Rev. Cubana de Hematol. Inmunologia y Hemoterapia.

[48] Woo MS (2020). Frequent neurocognitive deficits after recovery from mild COVID-19. Brain Commun.

[49] Blomberg B (2021). Long COVID in a prospective cohort of home-isolated patients. Nat Med.

[50] Hampshire, A. *et al.* Cognitive deficits in people who have recovered from COVID-19 relative to controls: An N=84,285 online study. *medRxiv* 2020.10.20.20215863 (2020).

[51] Hellmuth J (2021). Persistent COVID-19-associated neurocognitive symptoms in non-hospitalized patients. J Neurovirol.

[52] Henneghan, A. M. *et al.* Describing cognitive function and psychosocial outcomes of COVID-19 survivors. *J. Am. Assoc. Nurse. Pract.* Publish Ah, (2021). 10.1097/JXX.0000000000000647 PMC8882196 34469360

[53] Leth, S. *et al.* Persistent symptoms in patients recovering from COVID-19 in Denmark. *Open Forum Infect. Dis.* **8**, (2021). 10.1093/ofid/ofab042 PMC7928683 33875970

[54] Patel, R. *et al.* COVID-19 and Cognitive impairment: Severity, evolution, and functional impact during inpatient rehabilitation. *medRxiv* 2021.03.15.21253637 (2021).

[55] Pilotto, A. *et al.* COVID-19 severity impacts on long-term neurological manifestation after hospitalisation. *medRxiv* 2020.12.27.20248903 (2021).

[56] Pinnock, F. *et al.* Neurocognitive outcome following recovery from severe acute respiratory syndrome-coronavirus-1 (SARS-CoV-1). *J. Int. Neuropsychol. Soc.* 1–11 (2021) 10.1017/S1355617721001107. 10.1017/S1355617721001107 34488921

[57] Ramani C (2021). Post-ICU COVID-19 outcomes: A case series. Chest.

[58] Venturelli S (2021). Surviving COVID-19 in Bergamo province: A post-acute outpatient re-evaluation. Epidemiol Infect.

[59] Stern Y (2020). Whitepaper: Defining and investigating cognitive reserve, brain reserve, and brain maintenance. Alzheimer’s Dementia.

[60] Stern Y (2009). Cognitive reserve. Neuropsychologia.

[61] Boals A, Banks JB (2020). Stress and cognitive functioning during a pandemic: Thoughts from stress researchers. Psychol Trauma.

[62] Maloney EA, Sattizahn JR, Beilock SL (2014). Anxiety and cognition. *WIREs*. Cognit. Sci..

[63] Necho, M., Tsehay, M., Birkie, M., Biset, G. & Tadesse, E. Prevalence of anxiety, depression, and psychological distress among the general population during the COVID-19 pandemic: A systematic review and meta-analysis. *Int. J. Soc. Psychiatry* **67**, 892–906 Preprint at 10.1177/00207640211003121 (2021). 10.1177/00207640211003121 33794717

[64] Miners S, Kehoe PG, Love S (2020). Cognitive impact of COVID-19: looking beyond the short term. Alzheimers Res Ther.

[65] Hopkins RO (1999). Neuropsychological sequelae and impaired health status in survivors of severe acute respiratory distress syndrome. Am J Respir Crit Care Med.

[66] Gorelick PB (2010). Role of inflammation in cognitive impairment: Results of observational epidemiological studies and clinical trials. Ann N Y Acad Sci.

[67] Sartori AC, Vance DE, Slater LZ, Crowe M (2012). The impact of inflammation on cognitive function in older adults: Implications for healthcare practice and research. J. Neurosci. Nurs..

[68] Jung RG (2021). Methodological quality of COVID-19 clinical research. Nat Commun.

[69] Tombaugh TN, McIntyre NJ (1992). The mini-mental state examination: A comprehensive review. J Am Geriatr Soc.

[70] Pangman VC, Sloan J, Guse L (2000). An examination of psychometric properties of the mini-mental state examination and the standardized mini-mental state examination: Implications for clinical practice. Appl. Nurs. Res..

