# Supplementary material for: Neuropsychological deficits in patients with persistent COVID-19 symptoms: a systematic review and meta-analysis
Source: Sci Rep. 2023 Jun 26;13:10309. doi: 10.1038/s41598-023-37420-6 (PMC10293265; doi:10.1038/s41598-023-37420-6)
Supplement: Supplementary file 1 — Supplementary Information. [file 41598_2023_37420_MOESM1_ESM.pdf]

Title: Neuropsychological deficits in patients with persistent COVID-19 symptoms: A systematic review and meta-analysis.

Authors: [1]Saioa Sobrino-Relaño, [1]Yolanda Balboa-Bandeira., [1]Javier Peña, [1]Naroa Ibarretxe-Bilbao., [1]Leire Zubiaurre-Elorza., [1*]Natalia Ojeda.

Affiliation: [1]Department of Psychology, Faculty of Health Sciences, University of Deusto, Bilbao (Spain).

***Corresponding author:** Department of Psychology, Faculty of Health Sciences, University of Deusto, Av. de las Universidades, 24, Bilbao, Spain 48007. E-mail address: nojeda@deusto.es Telephone: +34 944 139 000 Ext: 2702

Supplementary material: Risk of bias assessment and search strategy details

Figure S1

Summary of the risk of bias for each included study [1,2].

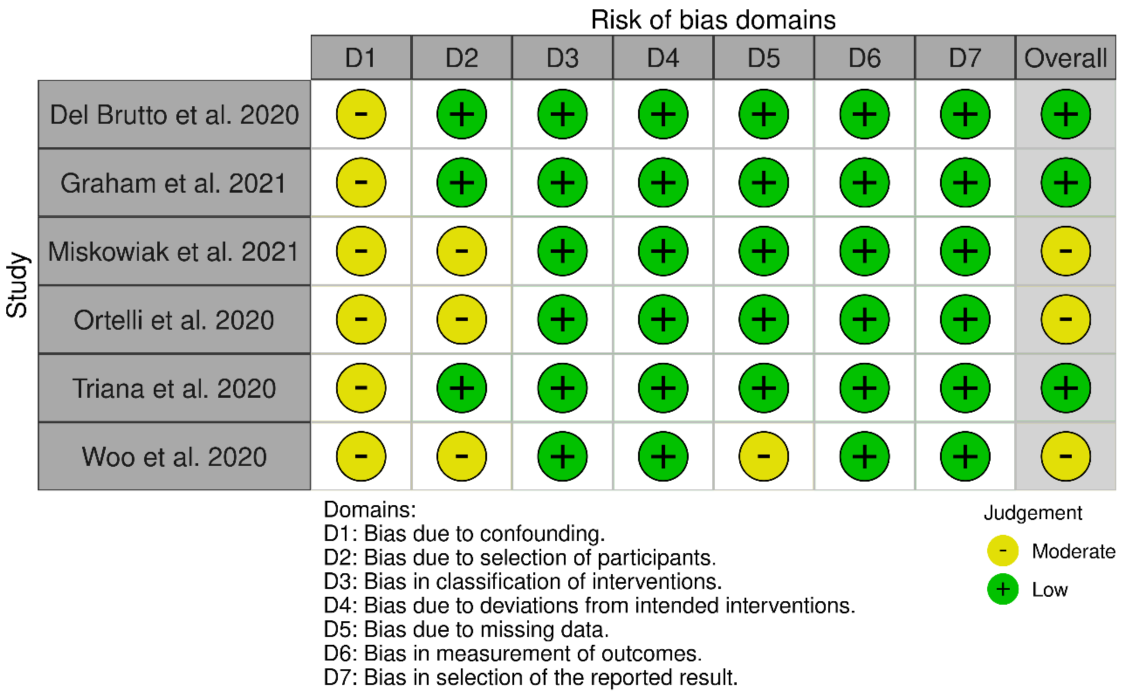

Table S1.

Search strategy key words used combinations on Pubmed, CENTRAL, Scopus and Web of Science databases.

**CONCEPT 1**		**CONCEPT 2**				**Number of obtained results (1602)**
(SARS-CoV-2 OR coronavirus OR COVID-19 OR COVID OR severe acute respiratory syndrome 2)	**AND**	*(long-COVID OR post-COVID syndrome OR post-acute COVID-19 syndrome OR long-term symptoms)*				1073
**CONCEPT 1**	**AND**	**CONCEPT 2**		**CONCEPT 3**		**N**
(SARS-CoV-2 OR coronavirus OR COVID-19 OR COVID OR severe acute respiratory syndrome 2)	**AND**	*(long-COVID OR post-COVID syndrome OR post-acute COVID-19 syndrome OR long-term symptoms OR persistent COVID-19)*	**AND**	*(neuropsychology OR cognition* OR psychology)*		437
(SARS-CoV-2 OR coronavirus OR COVID-19 OR COVID OR severe acute respiratory syndrome 2)	**AND**	*(long-COVID OR post-COVID syndrome OR post-acute COVID-19 syndrome OR long-term symptoms)*	**AND**	*(neuropsychological deficits* OR cognitive deficits* OR cognitive function*)*		77
**CONCEPT 1**		**CONCEPT 2**		**CONCEPT 3**	**CONCEPT 4**	**N**
(SARS-CoV-2 OR coronavirus OR COVID-19 OR COVID OR severe acute respiratory syndrome 2)	**AND**	*(long-COVID OR post-COVID syndrome OR post-acute COVID-19 syndrome OR long-term symptoms)*	**AND**	*AND (neuropsychological deficits* OR cognitive deficits* OR cognitive function*)*	(“Neuropsychological evaluation*” OR “Neuropsychological assessment*” OR “neuropsychological battery*” OR “cognitive evaluation*” OR “cognitive assessment*” OR “cognitive test*” OR “cognitive screening”)	15

References

1. Sterne, J. A. *et al.* ROBINS-I: A tool for assessing risk of bias in non-randomised studies of interventions. *BMJ* **355**, 4–10 (2016).
2. McGuinness, L. & Higgins, J. Risk-of-bias VISualization (robvis): An R package and Shiny web app for visualizing risk-of-bias assessments. 1–7 (2020) doi:https://doi.org/10.1002/jrsm.1411.
